# Tuning the electromagnetic and magnetic response of SrFe_12_O_19_ by Dy–Ce co-substitution for X-band microwave absorption

**DOI:** 10.1039/d6ra02983c

**Published:** 2026-07-03

**Authors:** Shreepad S. Atkare, S. B. Deshmukh, Vinod N. Dhage, Akash V. Fulari, Heba Taha Mohmmed Abdelghani, Sagar E. Shirsath, Maheshkumar L. Mane

**Affiliations:** a Shikshan Maharshi Guruvarya R. G. Shinde Mahavidyalaya Paranda Osmanabad MS India mane.maheshkumar@hotmail.com; b Department of Physics, Ramkrishna Paramhans College Dharashiv MS India; c Advanced Materials and Nanotechnology Research Laboratory, Department of Physics, MES Abasaheb Garware College Pune 411004 MS India; d Symbiosis Centre for Research and Innovation, Symbiosis International (Deemed University) Pune MS India; e Department of Exercise Physiology, College of Sport Sciences and Physical Activity, King Saud University P. O. Box 2454 Riyadh 11451 Saudi Arabia habdulghani@ksu.edu.sa; f School of Materials Science and Engineering, University of New South Wales Sydney NSW 2052 Australia s.shirsath@unsw.edu.au shirsathsagar@hotmail.com

## Abstract

Dy–Ce co-substituted strontium hexaferrite, SrDy_*x*_Ce_*x*_Fe_12−2*x*_O_19_ (*x* = 0.00–0.10), was synthesized by the sol–gel auto-combustion method and investigated as an X-band microwave attenuation material. Structural analysis confirmed the formation of the M-type hexaferrite phase for most compositions, with only minor lattice distortion after substitution; however, a secondary α-Fe_2_O_3_ phase appeared at *x* = 0.06, indicating a local limit to phase stability. Microstructural studies revealed platelet-like grains characteristic of hexaferrites and showed progressive grain growth with increasing Dy–Ce content. Magnetic measurements demonstrated a composition-dependent response, with the highest saturation magnetization obtained for *x* = 0.04, while excessive substitution led to deterioration of magnetic performance. Electromagnetic measurements showed that Dy–Ce co-substitution significantly improved microwave attenuation relative to pristine SrFe_12_O_19_. The optimized composition, *x* = 0.04, exhibited a minimum reflection loss of −25.26 dB at 9.6 GHz for a thickness of 3 mm, together with enhanced attenuation constant, improved impedance matching, and simultaneous increases in dielectric and magnetic loss contributions. Cole–Cole analysis suggested distributed dielectric relaxation behavior in the substituted samples. Although the total EMI shielding effectiveness remained modest, the shielding response was predominantly absorption driven. These results demonstrate that moderate Dy–Ce co-substitution effectively tunes the structure–property–performance relationship of SrFe_12_O_19_ and makes it a promising absorption-oriented microwave attenuator in the X-band region.

## Abstract 2

Dy–Ce co-substituted strontium hexaferrite, SrDy_*x*_Ce_*x*_Fe_12−2*x*_O_19_ (*x* = 0.00–0.10), was synthesized by the sol–gel auto-combustion method and investigated as an X-band microwave attenuation material.

## Graphical abstract

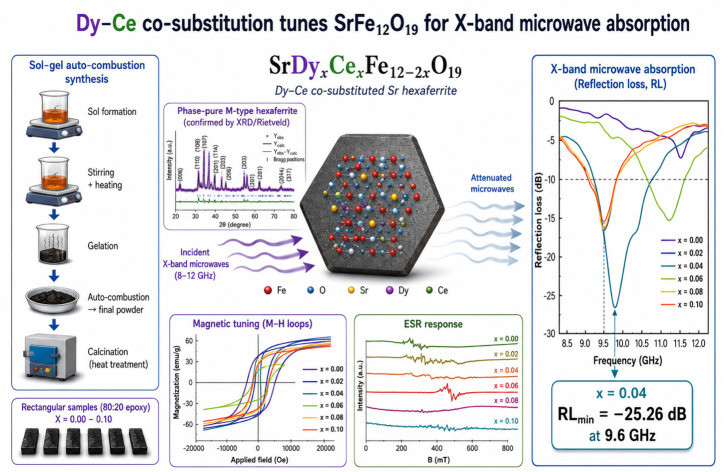

## 1. Introduction

The rapid expansion of wireless communication systems, radar technologies, and high-frequency electronic devices has intensified concerns about electromagnetic pollution and electromagnetic interference (EMI). Unwanted electromagnetic radiation can degrade signal quality, reduce device reliability, and interfere with the operation of sensitive electronic systems.^1–3^ As a result, there is growing interest in materials that can attenuate incident electromagnetic waves efficiently, particularly through absorption rather than simple reflection, since absorption-dominant behavior helps minimize secondary electromagnetic pollution.^4–7^

Oxide ceramics are considered to be one of the important materials for these kinds of versatile applications.^8,9^ Among magnetic ceramics, M-type strontium hexaferrite (SrFe_12_O_19_) has attracted considerable attention for high-frequency electromagnetic applications because of its high chemical stability, large magnetocrystalline anisotropy, significant coercivity, and intrinsic magnetic loss in the gigahertz regime.^10,11^ These characteristics make SrFe_12_O_19_ a promising candidate for microwave attenuation and related EMI-control applications.^12^ However, pristine strontium hexaferrite often exhibits limited microwave absorption performance due to inadequate dielectric loss, insufficient impedance matching, narrow effective absorption characteristics, and restricted dielectric tunability.^6,13,14^ Therefore, compositional modification has become an important strategy for improving its electromagnetic response, particularly through cation substitution at the Fe^3+^ sites.^15,16^

Rare-earth substitution has been widely explored as an effective route for tuning the structural, magnetic, and dielectric properties of ferrite-based materials.^17–21^ In this context, Dy and Ce are of particular interest because they can influence complementary loss mechanisms. Dy^3+^ substitution has been reported to enhance magnetocrystalline anisotropy and magnetic loss processes that are relevant to microwave attenuation.^22,23^ Ce-containing substitutions, on the other hand, have been associated with improved polarization behavior and dielectric-loss-related contributions, which can assist electromagnetic attenuation when appropriate impedance matching is achieved.^24,25^ Such dual tuning of magnetic and dielectric responses is important for developing ferrite absorbers with balanced loss characteristics.

Previous studies have shown that Dy substitution can improve the microwave absorption behavior of ferrite systems such as Mn–Zn ferrites and Ba–Ca hexaferrites.^26,27^ Likewise, Ce substitution has been reported to enhance dielectric polarization, impedance matching, and absorption-related performance in hexaferrite materials.^6,28,29^ Nevertheless, most of the available literature has focused on single-ion substitution or on barium-based hexaferrites, while studies on Dy–Ce co-substituted strontium hexaferrite remain limited. In particular, a systematic investigation that correlates phase evolution, microstructure, magnetic behavior, and X-band electromagnetic attenuation in Dy–Ce co-substituted SrFe_12_O_19_ is still needed.

Motivated by this gap, the present work investigates Dy–Ce co-substituted strontium hexaferrite, SrDy_*x*_Ce_*x*_Fe_12−2*x*_O_19_, prepared by the sol–gel auto-combustion route. The study aims to examine how Dy–Ce co-substitution affects the crystal structure, morphology, magnetic properties, and electromagnetic parameters of SrFe_12_O_19_, and how these changes influence microwave attenuation behavior in the X-band region. Reflection loss, shielding contributions, attenuation characteristics, dielectric and magnetic losses, and impedance matching are analyzed to identify the composition that offers the most favorable balance of properties. Through this structure–property–performance correlation, the work seeks to clarify the role of Dy–Ce co-substitution in tailoring strontium hexaferrite for microwave absorption-oriented electromagnetic applications.

## 2. Methods and materials

### 2.1 Synthesis of SrDy_*x*_Ce_*x*_Fe_12−2*x*_ O_19_ hexaferrite *via* sol–gel auto combustion method

Dy–Ce co-substituted strontium hexaferrite with nominal composition SrDy_*x*_Ce_*x*_Fe_12−2*x*_O_19_ (*x* = 0.00, 0.02, 0.04, 0.06, 0.08, 0.1) was synthesized using the sol–gel auto-combustion method.^30^ The starting precursors were strontium nitrate Sr(NO_3_)_2_, dysprosium nitrate (Dy(NO_3_)_3_·*x*H_2_O), cerium nitrate (Ce(NO_3_)_3_·6H_2_O), ferric nitrate (Fe(NO_3_)_3_·9H_2_O), and citric acid (C_6_H_8_O_7_·H_2_O) as the complexing/fuel agent. The stoichiometric quantities used for preparing 10 g of each composition are listed in Table S1.

For each batch, the required amounts of the metal nitrate precursors were weighed according to the nominal composition and dissolved in 200 mL of distilled water under continuous stirring to obtain a homogeneous solution. Citric acid was then introduced to promote complexation of the metal ions and to facilitate gel formation during subsequent heating. The pH of the mixed precursor solution was adjusted to 8 by the dropwise addition of ammonia solution.

The resulting solution was heated under continuous stirring on a magnetic stirrer-hotplate until solvent evaporation led to gel formation. With further heating, the gel gradually transformed into a viscous xerogel, followed by spontaneous auto-combustion due to the exothermic redox reaction between nitrate groups acting as oxidizers and the organic fuel. This combustion process yielded a voluminous, fluffy precursor powder. The as-burnt powder was then calcined/sintered in a muffle furnace at 1100 °C for 6 h to improve crystallinity and promote the formation of the hexaferrite phase.

### 2.2 Characterizations

**Fig. 1:**
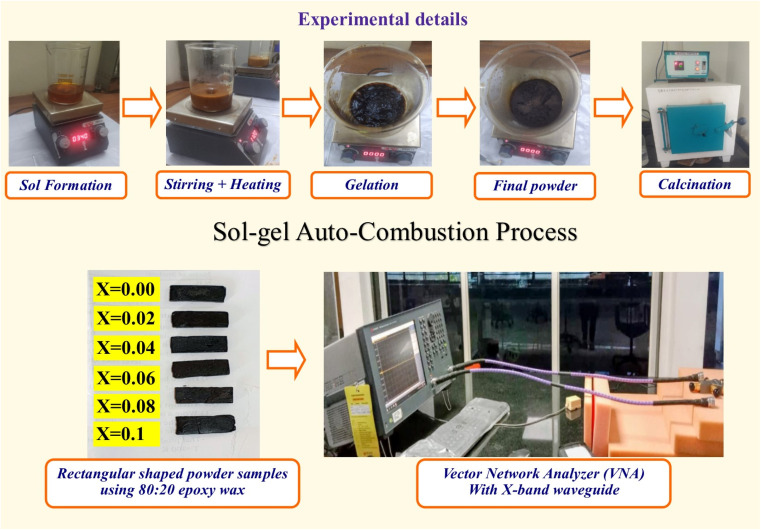
Sol–gel auto-combustion process details for preparation of Dy–Ce substituted strontium hexaferrite with experimental setup for measurement of electromagnetic parameters.

An X-ray diffractometer (Rigaku D/Max2500) with Cu-Kα radiation (wavelength of *λ* = 1.5406 Å) was used to characterize the crystal structure. Using a Bruker model instrument, the FTIR spectra were acquired in the 400–4000 cm^−1^ region. ESR spectra in the X-band frequency region were recorded at room temperature using a JEOL-24 electron spin resonance (ESR) spectrometer. Grain morphology and material structure were assessed at the atomic resolution level using high-voltage (20 kV) FE-SEM imaging (Fei, Nova Nanosem 450 Model) and transmission electron microscopy (HRTEM) (Jeol F200 Model) respectively. Magnetic properties were measured using a microscence vibrating sample magnetometer (VSM) with a 1.5 tesla magnetic field. The microwave absorption properties of a material made of epoxy wax at an 80 : 20 mass ratio and shaped like a rectangle were measured in the X-band frequency range (8 to 12 GHz) using a vector network analyzer (ENA E5063A model). The detailed process of sol–gel Auto-combustion for preparation of Dy–Ce substituted strontium hexaferrite with experimental setup for measurement of electromagnetic parameters is illustrated in Fig. 1.

## 3. Results and discussion

### 3.1 Structural analysis

**Fig. 2:**
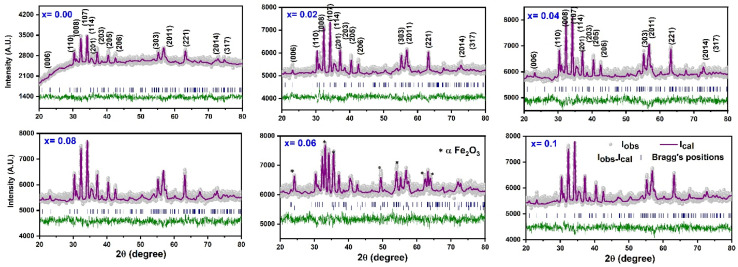
Rietveld refined XRD patterns of all the samples of SrDy_*x*_Ce_*x*_Fe_12−2*x*_O_19_.

The Rietveld-refined X-ray diffraction patterns of Dy–Ce co-substituted strontium hexaferrite, SrDy_*x*_Ce_*x*_Fe_12−2*x*_O_19_ (*x* = 0.00, 0.02, 0.04, 0.06, 0.08, and 0.10), are presented in Fig. 2. The refinement was carried out using the FullProf Suite, employing a pseudo-Voigt profile function for peak fitting and linear interpolation between selected background points for background modeling. During the refinement, the scale factor, lattice parameters, and profile-related parameters were refined simultaneously.^31^ The crystallographic information file associated with COD entry 1 006 000 was used as the structural input model for phase identification and refinement. The refinement quality indicators, including the profile factor (*R*_p_), weighted profile factor (*R*_wp_), expected profile factor (*R*_e*x*p_), and goodness of fit (*χ*^2^), are summarized in Table 1.

**Table 1:** Lattice parameter (*c* and *a*), *c*/*a* ratio, volume of unit cell (V), X-ray density (*d*_X_), *R*-factors (*R*_p_, *R*_wp_ and *R*_exp_), goodness fit index (*χ*^2^) for Dy–Ce substituted SrFe_12_O_19_

Comp. (*x*)	Lattice parameter (Å)		*c*/*a* (Å)	*V* (Å^3^)	*d*_X_ (g cm^−3^)	*R*_p_	*R*_wp_	*R*_e*x*p_	*χ* ^2^
	*a*	*c*							
0.00	5.881	23.074	3.923	691.10	5.102	76.3	51.1	42.41	1.45
0.02	5.882	23.063	3.922	691.01	5.121	59.1	38.1	30.16	1.60
0.04	5.879	23.066	3.923	690.39	5.144	58.6	36.9	30.02	1.51
0.06	5.877	23.066	3.925	689.92	5.166	57.2	38.3	32.23	1.41
0.08	5.880	23.065	3.923	690.60	5.179	56.0	35.9	28.77	1.56
0.1	5.883	23.070	3.921	691.45	5.191	56.2	35.3	28.58	1.53

The refined diffraction profiles show good agreement between the observed and calculated patterns, indicating that the dominant phase in all compositions corresponds to M-type strontium hexaferrite with hexagonal crystal symmetry and space group *P*6_3_/*mmc*.^32,33^ The *χ*^2^ values in the range of 1.41–1.60 further support the reliability of the refinement. The presence of well-defined diffraction peaks corresponding to characteristic planes such as (110), (107), (114), (203), (206), and (217) confirms the formation of a highly crystalline hexaferrite phase.^34^ For most compositions, no additional peaks corresponding to CeO_2_, Dy_2_O_3_, or α-Fe_2_O_3_ were detected, suggesting effective phase formation within the detection limit of XRD. However, for the composition *x* = 0.06, a secondary α-Fe_2_O_3_ phase was observed, indicating partial phase segregation at this substitution level.^35,36^ This behavior is in accordance with the low solubility of rare-earth ions in the SrFe_12_O_19_ lattice. This phase segregation observed, attributed to differences in ionic radius, valence state and site preference.^37^. The inclusion of α-Fe_2_O_3_ as a secondary phase in the refinement improved the fitting quality for this composition, further supporting its presence.

The lattice parameters obtained from the Rietveld refinement are listed in Table 1. The undoped sample (*x* = 0.00) shows lattice constants of *a* = 5.881 Å and *c* = 23.074 Å, which are in good agreement with reported values for SrFe_12_O_19_.^38^ Upon Dy–Ce co-substitution, only small variations in the lattice parameters are observed, with a ranging from 5.877 to 5.883 Å and *c* varying from 23.063 to 23.070 Å. These modest changes indicate that the magnetoplumbite structure is largely retained after substitution, the observed changes of the lattice parameters are in agreement with local lattice distortion linked with the incorporation of Dy–Ce.^39–41^

The *c*/*a* ratio remains nearly constant in the range of 3.921–3.925, indicating that the overall hexagonal symmetry is preserved across the substitution series.^42^ The unit cell volume was calculated using the standard relation for a hexagonal structure^43^

1 *V* = 0.866*a*^2^*c*

The X-ray density was determined using the relation^44^2
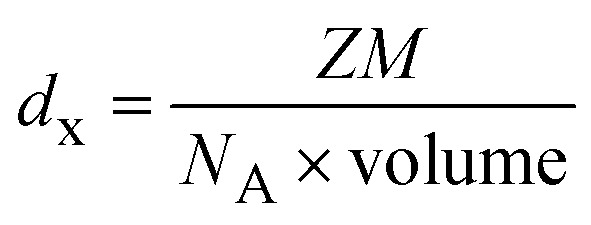
where *N*_A_ = 6.023 × 10^23^, *Z* is the number of formula units per unit cell, and *M* is the molecular weight.

The unit cell volume shows a slight decrease from 691.10 Å^3^ to 689.92 Å^3^, followed by a small increase to 691.45 Å^3^ at higher substitution levels. This trend indicates that Dy–Ce incorporation induces only limited structural distortion. The observed variation is consistent with cation substitution and redistribution within the hexaferrite lattice.^6^ At the same time, the X-ray density increases from 5.102 to 5.191 g cm^−3^, which can be attributed primarily to the increase in molecular weight associated with rare-earth substitution.^45^

In the present study, the oxidation state of cerium was not directly determined. However, our earlier XPS study of Dy–Ce substituted barium hexaferrite indicated the presence of Ce^3+^ and Ce^4+^ species, which suggests that the cerium in rare earth substituted hexaferrites has mixed valency.^11^ Based on this observation and related literature reports, similar mixed-valence states may also be present in the present SrDy_*x*_Ce_*x*_Fe_12−2*x*_O_19_ system. In such cases charge compensation can occur by oxygen-vacancy formation and/or partial Fe^3+^/Fe^2+^ redistribution, which can contribute to local lattice distortion and defect formation.

### 3.2 Spectral analysis

#### 3.2.1 FT-IR spectroscopy

**Fig. 3:**
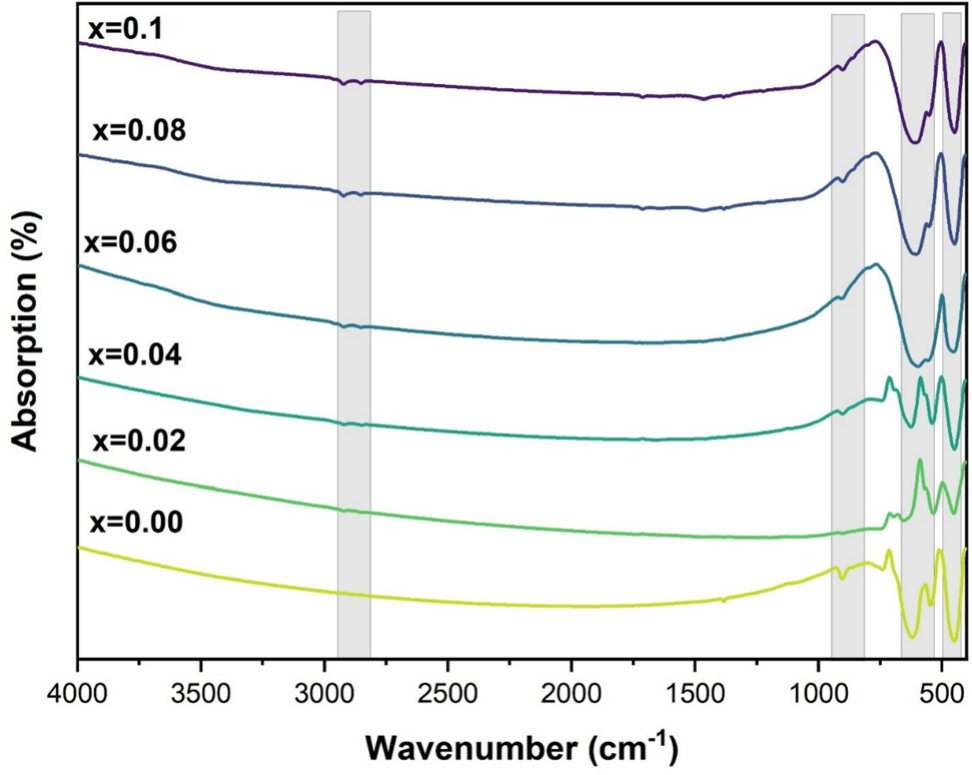
FTIR spectra of Dy–Ce substituted SrFe_12_O_19_ of all compositions (*x* = 0.00, 0.02, 0.04, 0.06, 0.08, and 0.10).

The FT-IR spectra of Dy–Ce co-substituted strontium hexaferrite recorded in the 400–4000 cm^−1^ region are presented in Fig. 3. All compositions (*x* = 0.00, 0.02, 0.04, 0.06, 0.08, and 0.10) exhibit broadly similar spectral features, indicating that Dy–Ce incorporation does not significantly alter the fundamental vibrational characteristics of the M-type hexaferrite framework. This overall spectral similarity is consistent with the XRD results, which also suggest retention of the hexagonal magnetoplumbite structure after substitution.

Two characteristic absorption bands are observed in the low-wavenumber region, located at approximately 441–445 cm^−1^ and 550–600 cm^−1^. These bands are assigned to Fe–O stretching vibrations associated with tetrahedral and octahedral coordination environments, respectively, and are typical of M-type strontium hexaferrite.^36,46^ The persistence of these two principal ferrite bands across the entire composition range supports the formation of the hexaferrite phase and suggests that the basic Fe–O polyhedral network remains intact after Dy–Ce substitution. Also a weak band around 950–1000 cm^−1^ is observed which is in agreement with C–O stretching vibrations attributed to residual carbon containing species from the sol–gel auto-combustion process.^47^

With increasing Dy–Ce content, small shifts in band position together with changes in relative intensity are observed. These variations reflect changes in the local bonding environment upon Dy–Ce substitution. The observed shifts are in agreement with changes in metal–oxygen force constants due to differences in ionic radius, mass and valence state of the substituent ions and Fe^3+^.^48^ Rather than indicating a major structural transformation, these spectral changes are more reasonably interpreted as evidence of local lattice distortion within an otherwise preserved hexaferrite framework. In this sense, the FT-IR results support the view that Dy–Ce substitution affects the short-range vibrational environment of the Fe–O sublattice while maintaining the overall structural identity of SrFe_12_O_19_.

### 3.3 Morphological properties

#### 3.3.1 Field emission scanning electron microscopy (FE-SEM) analysis

**Fig. 4:**
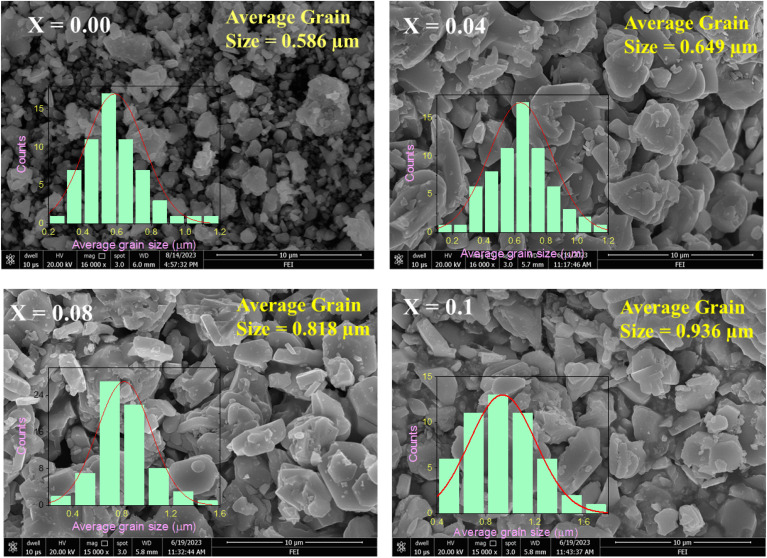
FE-SEM images and particle size distribution of SrDy_*x*_Ce_*x*_Fe_12−2*x*_O_19_ (*x* = 0.00, 0.04, 0.08, 0.1).

The FE-SEM micrographs of Dy–Ce co-substituted strontium hexaferrite for selected compositions are presented in Fig. 4, together with the corresponding particle size distribution histograms. The micrographs reveal that all samples are composed of aggregated grains with morphology characteristic of hexaferrite systems. The undoped sample (*x* = 0.00) exhibits relatively fine and irregularly shaped grains with noticeable agglomeration, giving an average grain size of approximately 0.586 µm.^49^ Upon Dy–Ce co-substitution, a gradual evolution in grain morphology is observed. For *x* = 0.04, the particles become more distinct and exhibit improved intergranular contact, with a moderate increase in average grain size to about 0.649 µm. A further increase in substitution to *x* = 0.08 leads to thicker and more uniformly distributed grains, with an average size of about 0.818 µm. At the highest investigated substitution level (*x* = 0.10), the sample shows comparatively larger and better-developed grains with smoother surfaces, and the average grain size increases to approximately 0.936 µm.

The observed morphology, marked by slightly agglomerated hexagonal platelet-like grains, is consistent with the expected microstructural features of M-type hexaferrites.^50,51^ The progressive increase in grain size with Dy–Ce content suggests that rare-earth substitution influences the sintering behavior and grain growth kinetics of the system. The behavior is associated with changes in lattice strain, local diffusion conditions and grain boundary mobility induced by Dy–Ce substitution at or near Fe containing lattice sites.^52,53^ From a functional viewpoint, such microstructural evolution is important because grain size and particle connectivity can affect both magnetic response and high-frequency electromagnetic attenuation by modifying interfacial polarization, magnetic domain behavior, and the effective propagation pathway of incident microwaves.

#### 3.3.2 High-resolution transmission electron microscopy (HR-TEM) analysis

**Fig. 5:**
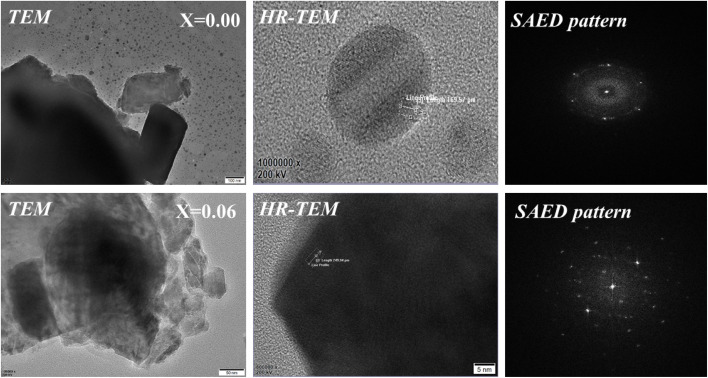
Transmission electron microscope (TEM) image, corresponding high-resolution (HRTEM) images, selected area electron diffraction (SAED) pattern of SrDy_*x*_Ce_*x*_Fe_12−2*x*_O_19_ nanoparticles (*x* = 0.00 & *x* = 0.06).

To further examine the microstructure at higher spatial resolution, HR-TEM analysis was carried out for the undoped sample (*x* = 0.00) and the substituted sample (*x* = 0.06), as shown in Fig. 5. The TEM image of the undoped composition reveals plate-like particles with moderate agglomeration, in agreement with the morphology expected for hexaferrite materials.^42^ Such agglomeration is commonly observed in magnetic ferrites and can be attributed to interparticle magnetic dipole–dipole interactions in combination with the high-temperature calcination process.^54^ In contrast, the substituted sample (*x* = 0.06) exhibits comparatively larger and denser particles, indicating that Dy–Ce incorporation promotes grain development and particle coalescence, consistent with the FE-SEM observations.

The HR-TEM images of both samples display clear and well-resolved lattice fringes, confirming the crystalline nature of the synthesized particles. The measured interplanar spacings are consistent with the characteristic planes of M-type strontium hexaferrite, supporting the phase analysis obtained from XRD. In addition, the selected-area electron diffraction (SAED) patterns exhibit concentric diffraction rings rather than isolated spots, indicating the polycrystalline nature of the particles.^55^ Taken together, the TEM, HR-TEM, and SAED observations confirm that the synthesized materials possess a crystalline hexaferrite structure with platelet-like morphology, while also showing that Dy–Ce substitution modifies particle growth and compactness at the nanoscale. These microstructural changes are in good agreement with the observed composition-dependent variations in magnetic and microwave attenuation behavior discussed in the following sections.

### 3.4 Magnetic properties

**Fig. 6:**
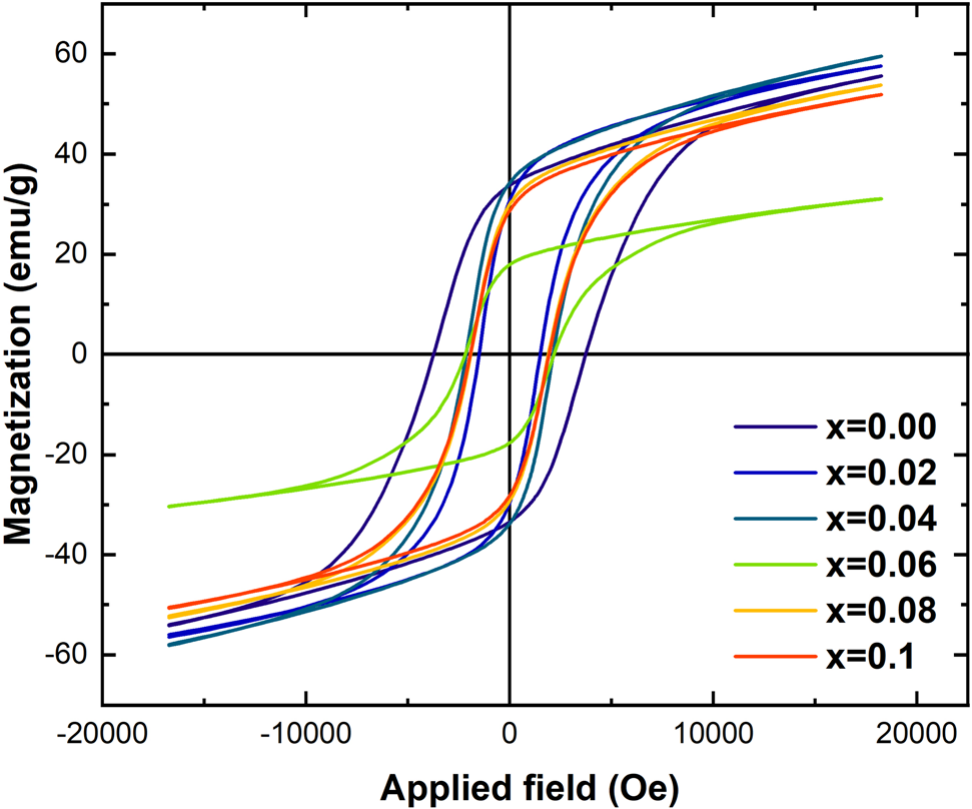
The magnetic hysteresis behavior of all samples of SrDy_*x*_Ce_*x*_Fe_12−2*X*_O_19_.

The room-temperature magnetic hysteresis (*M*–*H*) loops of Dy–Ce co-substituted strontium hexaferrite, SrFe_12−2*x*_(Dy,Ce)_*x*_O_19_ (*x* = 0.00–0.10), are shown in Fig. 6.

All compositions exhibit well-defined hysteresis behavior, confirming the ferrimagnetic character typical of M-type hexaferrites.^56^ The magnetic parameters derived from the hysteresis loops, including saturation magnetization (*M*_s_), remanent magnetization (*M*_r_), coercive field (*H*_c_), squareness ratio (*M*_r_/*M*_s_), and Bohr magneton number (*n*_B_), are summarized in Table 2.

**Table 2:** Magnetic parameters for Dy–Ce substituted SrFe_12_O_19_

Comp. (*x*)	*M*_s_ (emu g^−1^)	*M*_r_ (emu g^−1^)	*H*_c_ (Oe)	*M*_r_/*M*_s_	*n*_B_ (*µ*_B_)
0.00	55.54	33.76	3735	0.61	10.56
0.02	57.03	30.68	1498	0.54	10.88
0.04	59.50	34.24	2156	0.58	11.39
0.06	30.73	17.82	2181	0.58	5.90
0.08	53.76	29.93	_19_08	0.56	10.37
0.1	51.29	28.68	_19_35	0.56	9.93

A composition-dependent variation in saturation magnetization is observed upon Dy–Ce co-substitution.^57,58^ The undoped sample shows an *M*_s_ value of 55.54 emu g^−1^, which increases gradually with substitution and reaches a maximum of 59.50 emu g^−1^ at *x* = 0.04. This improvement suggests that the ferrimagnetic ordering is improved at moderate substitution levels. The increase in magnetization observed is consistent with a rearrangement of cations and the modified exchange interactions in the hexaferrite lattice.^59,60^ At this level of substitution, the structural stability of the M-type phase is substantially preserved, consistent with an efficient magnetic coupling. However, at *x* = 0.06, the saturation magnetization decreases sharply to 30.73 emu g^−1^. This pronounced drop is consistent with the XRD results, which indicate the appearance of a secondary α-Fe_2_O_3_ phase. Since α-Fe_2_O_3_ contributes much less to the net magnetization than the ferrimagnetic hexaferrite phase, its formation is expected to dilute the overall magnetic response and weaken effective magnetic coupling in the material.^61–63^ For higher substitution levels (*x* = 0.08 and 0.10), *M*_s_ shows partial recovery, although the values remain below that of the undoped sample. This behavior suggests that too much substitution does not improve the magnetic ordering further. The decrease of the magnetization is in agreement with an increased degree of local disorder, changed exchange paths and a partial destruction of the magnetic sublattice arrangement.^64^

From a crystal-chemistry point of view, Dy^3+^ and Ce^3+^/Ce^4+^ substitution induces local distortions in the Fe–O framework due to differences in ionic radius and electronic configuration compared to Fe^3+^.^65^ Such substitution may alter Fe–O–Fe bond geometry and thus affect superexchange interactions between magnetic sublattices.^66,67^ The increase in the magnetization at *x* = 0.04 is consistent with a positive change in the magnetic exchange interactions, while higher levels of substitution introduce increased structural disorder and phase instability which adversely affects the long-range magnetic ordering.

The remanent magnetization follows a trend similar to that of *M*_s_, reaching a maximum value of 34.24 emu g^−1^ at *x* = 0.04. The simultaneous enhancement of *M*_s_ and *M*_r_ at this composition indicates that moderate Dy–Ce substitution improves magnetic alignment and stabilizes the ferrimagnetic state more effectively than either the undoped or more heavily substituted compositions.

In contrast, the coercive field decreases markedly after substitution. Pure SrFe_12_O_19_ exhibits the highest coercivity (3735 Oe), which is consistent with the strong uniaxial magnetocrystalline anisotropy of M-type hexaferrite. After Dy–Ce incorporation, Hc drops into the range of approximately 1900–2200 Oe. This reduction is consistent with the simultaneous effects of grain growth, lattice perturbation and magnetic anisotropy changes due to substitution at Fe containing sites.^40,68–70^ The FE-SEM results, which show progressive grain growth with increasing substitution, support this interpretation, since larger grains generally reduce resistance to magnetization reversal when compared with finer particles in highly anisotropic ferrites. Thus, the decrease in *H*_c_ suggests that Dy–Ce co-substitution softens the magnetic response relative to pristine SrFe_12_O_19_, even though the material remains magnetically hard in comparison with many soft ferrites.

The squareness ratio (*M*_r_/*M*_s_) decreases from 0.61 for the undoped sample to lower values for the substituted compositions, although it remains above 0.50 throughout the series. Such values indicate relatively high remanence and significant magnetic anisotropy.^71^ This behavior, instead of being a conclusive proof of a purely single-domain structure, suggests a significant magnetic anisotropy and an appreciable remanent magnetization. These characteristics are in agreement with the influence of the particle morphology and magnetic interactions in the synthesized hexaferrite system. The gradual reduction in squareness ratio with substitution is consistent with increasing structural and magnetic disorder.

The Bohr magneton number, *n*_B_, was calculated using the relation:^72^3
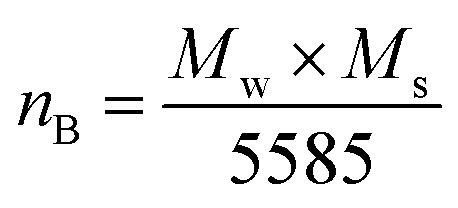
where *M*_w_ is the molecular weight of the corresponding composition. The calculated *n*_B_ values increase from 10.56 *µ*_B_ for *x* = 0.00 to 11.39 *µ*_B_ for *x* = 0.04, further supporting the improved magnetic response at moderate substitution. At *x* = 0.06, *n*_B_ decreases sharply to 5.90 *µ*_B_, in agreement with the strong reduction in saturation magnetization. This result reflects the deterioration of effective ferrimagnetic coupling at this composition, which is consistent with phase impurity and enhanced magnetic inhomogeneity.^73^

The magnetic results indicate that Dy–Ce co-substitution exerts a non-monotonic influence on the magnetic behavior of SrFe_12_O_19_. A moderate substitution level (*x* = 0.04) improves the ferrimagnetic response by increasing both saturation and remanent magnetization, whereas further substitution reduces magnetic performance due to phase instability, cation disorder, and weakened exchange interactions. At the same time, the observed decrease in coercivity suggests that Dy–Ce incorporation provides a route to tune the balance between magnetization and anisotropy, which is important for optimizing the high-frequency electromagnetic response discussed in later sections.

Electron spin resonance spectroscopy (ESR) is an analytical technique that measures a material's magnetic properties, such as magnetic interaction and the Lande *g*-factor, which gives information about homogeneity and crystallographic anisotropy by measuring the effective magnetic field experienced by unpaired electrons.^74^

**Fig. 7:**
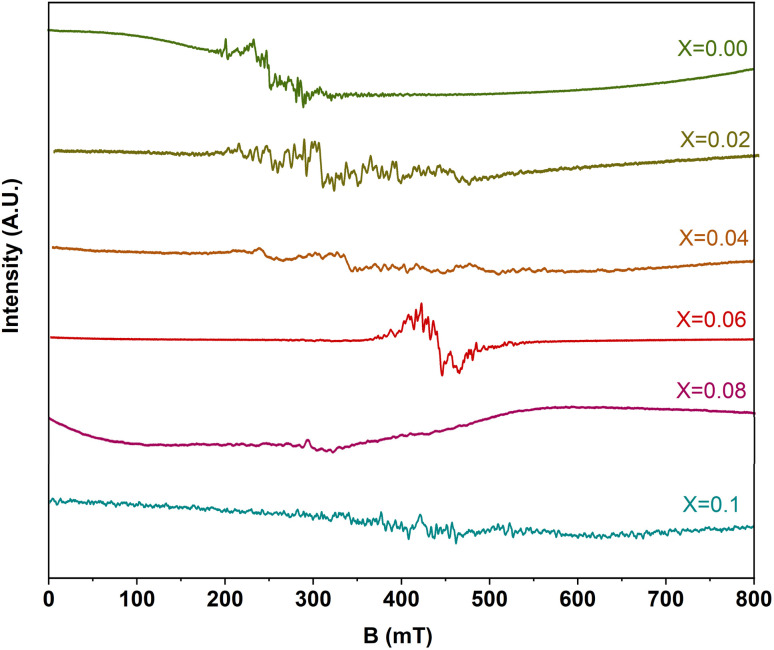
ESR spectra of Dy–Ce substituted SrFe_12_O_19_.

The X-band ESR spectra of SrDy_*x*_Ce_*x*_Fe_12−2*x*_O_19_ are shown in Fig. 7, and the extracted resonance parameters are summarized in Table 3. The spectra do not show a simple monotonic displacement with Dy–Ce substitution; instead, the resonance profile, linewidth, asymmetry and intensity change substantially with composition. This indicates that the ESR response is governed by a combination of ferrimagnetic resonance from the SrFe_12_O_19_ matrix, Fe^3+^-centered resonance environments, and substitution/phase-induced local magnetic fields, rather than by a single isolated paramagnetic center.^74,75^

**Table 3:** Quantitative CW-ESR parameters of SrDy_*x*_Ce_*x*_Fe_12−2*x*_O_19_ extracted from the first-derivative X-band ESR spectra, including positive and negative derivative extrema (*B*_ma*x*_ and *B*_min_), effective resonance field (*B*_r_), effective *g*-value (*g*_eff_), peak-to-peak linewidth (*Δ*_Hpp_), peak-to-peak intensity (*I*_pp_), relative double-integrated intensity, asymmetry factor, and linewidth-derived effective spin–spin relaxation time (*T*_2_)

*x*	*B*_max_ (mT)	*B*_min_ (mT)	*B*_r_ (mT)	*g*_eff_	*Δ*_Hpp_ (mT)	*I*_pp_ (a.u.)	Relative integrated intensity	Asymmetry factor	Effective *T*_2_ (ns)
0	233.6	290	251.2	2.7	56.4	1380	1	1.11	0.086
0.02	242.1	259	251.5	2.7	16.9	519	0.44	0.72	0.287
0.04	188.6	290.6	222.1	3.06	102.1	411	1.66	0.21	0.042
0.06	316.3	334	330.4	2.05	17.7	1772	0.54	1.06	0.361
0.08	423.2	241.1	348.4	1.95	182.1	506	3.53	0.64	0.037
0.1	257.8	273.2	258.2	2.63	15.4	1033	2.32	0.08	0.324

The effective resonance field (*B*_r_) was obtained from the zero-crossing position of the first-derivative ESR signal, while the peak-to-peak linewidth (*Δ*_Hpp_) was determined from the separation between the derivative extrema. The effective *g*-factor was estimated using the relation:^76^4
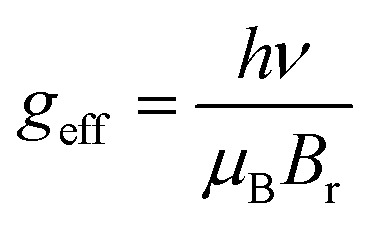
where *h*, *ν*, *µ*_B_ and *B*_r_ have their usual meanings. Since the spectra are broad and asymmetric, the calculated *g*_eff_ values should be treated as effective resonance parameters rather than intrinsic Landé *g*-factors. The variation of *g*_eff_ from 1.95 to 3.06 therefore reflects changes in the effective internal magnetic field experienced by the resonating species. In magnetically ordered hexaferrites, this field is strongly modified by magnetocrystalline anisotropy, exchange coupling, dipolar interactions and demagnetization effects, particularly in polycrystalline platelet-like grains.

The undoped SrFe_12_O_19_ sample shows a broad resonance centered at *B*_r_ = 251.2 mT with *Δ*_Hpp_ = 56.4 mT and *g*_eff_ ≈ 2.70. The shifted resonance position and broad linewidth are consistent with the hard ferrimagnetic character of M-type SrFe_12_O_19_, where the resonance condition is displaced from the free-electron position by strong internal anisotropy fields. With slight Dy–Ce substitution at *x* = 0.02, the linewidth decreases markedly to 16.9 mT, accompanied by an increase in the effective *T*_2_ value to 0.287 ns. This narrowing suggests that the dominant resonance contribution becomes more defined at low substitution, while the ferrimagnetic matrix remains structurally preserved, in agreement with the XRD results.

The *x* = 0.04 composition exhibits a distinctly broadened and asymmetric resonance response, with *Δ*_Hpp_ = 102.1 mT and an asymmetry factor of 0.21. This composition also shows the highest saturation magnetization and the strongest microwave absorption response among the investigated samples. The ESR broadening at *x* = 0.04 is therefore consistent with an enhanced distribution of local anisotropy fields and modified Fe–O–Fe exchange interactions introduced by moderate Dy–Ce incorporation. Such a resonance environment can contribute to magnetic loss in the X-band region, supporting the improved attenuation behavior observed from the reflection loss and attenuation constant analysis.^77^

A markedly different ESR feature appears for *x* = 0.06, where the resonance shifts to *B*_r_ = 330.4 mT with *g*_eff_ ≈ 2.05, *Δ*_Hpp_ = 17.7 mT and the highest peak-to-peak intensity. The *g*_eff_ value close to 2.0 indicates a stronger contribution from Fe^3+^-centered resonance environments. This assignment is consistent with the XRD evidence for an α-Fe_2_O_3_ secondary phase at the same composition. The simultaneous reduction of saturation magnetization at *x* = 0.06 further confirms that the ESR response is affected by phase segregation and altered magnetic coupling. Therefore, the large spectral shift at this composition is not attributed merely to lattice distortion, but to the emergence of a distinct Fe^3+^-rich resonance contribution associated with the secondary phase and modified ferrimagnetic exchange network.^78^

At higher substitution levels, the ESR response again becomes broad and heterogeneous. The *x* = 0.08 sample exhibits the largest linewidth, *Δ*_Hpp_ = 182.1 mT, together with the highest relative integrated intensity, indicating a wide distribution of resonance fields. This behavior is consistent with increased local magnetic heterogeneity caused by rare-earth-induced lattice distortion, redistribution of Fe-based magnetic environments, oxygen-vacancy-related charge compensation and modified exchange pathways. The *x* = 0.10 sample shows a narrower dominant linewidth than *x* = 0.08, but the line remains highly asymmetric, suggesting that multiple unresolved resonance environments still contribute to the overall signal.^79^

The spectral evolution can therefore be rationalized in terms of overlapping magnetic contributions. The broad low-field component is assigned to the collective ferrimagnetic resonance of the Fe^3+^-based SrFe_12_O_19_ matrix. The resonance feature near *g*_eff_ ≈ 2.0, most clearly observed for *x* = 0.06, is assigned to Fe^3+^-centered environments, with contribution from α-Fe_2_O_3_. The broad and asymmetric features at higher substitution are associated with substitution-induced distributions of anisotropy fields, defect-related magnetic environments and modified Fe–O–Fe exchange interactions. Ce^4+^ is not expected to contribute directly to the ESR signal because of its 4f_0_ configuration, while isolated Dy^3+^ or Ce^3+^ signals are not clearly resolved at room temperature, most likely due to strong relaxation and overlap with the dominant ferrimagnetic resonance background.

### 3.5. Microwave absorption properties

#### 3.5.1 Reflection loss and EMI shielding effectiveness

**Fig. 8:**
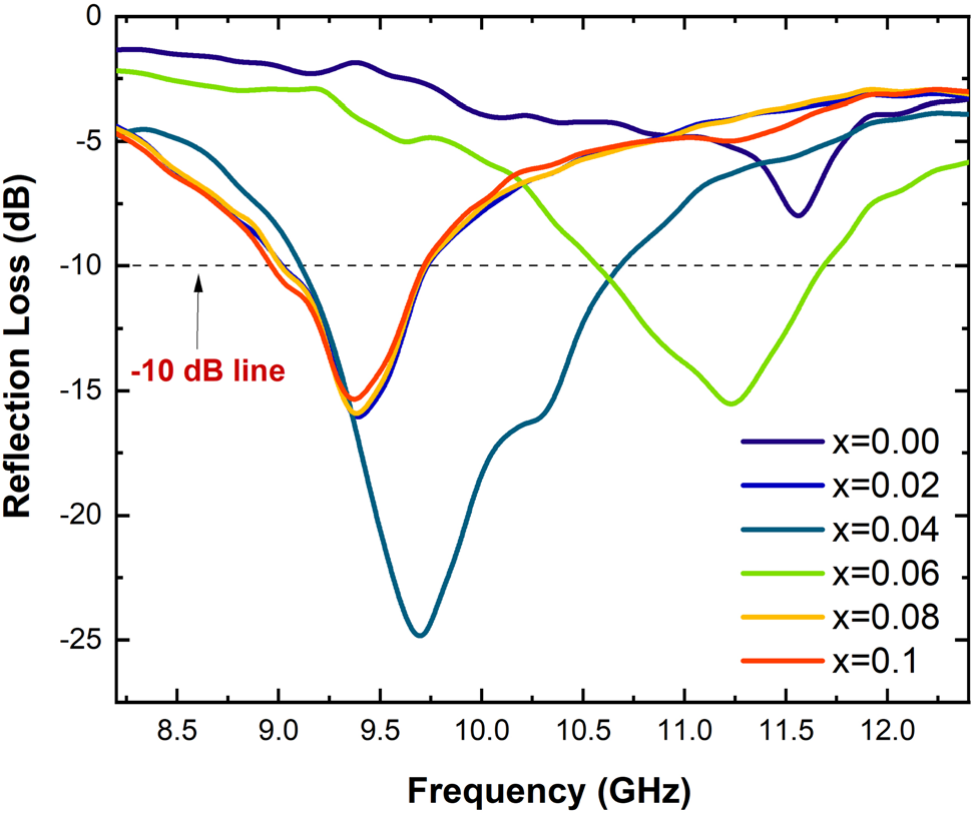
The variation of reflection loss *versus* frequency for SrDy_*x*_Ce_*x*_Fe_12−2*x*_O_19_.

The microwave absorption performance of SrDy_*x*_Ce_*x*_Fe_12−2*x*_O_19_ (*x* = 0.00, 0.02, 0.04, 0.06, 0.08, 0.1) was evaluated in X-band frequency range (8–12 GHz). The reflection loss spectra at fixed absorber thickness of 3 mm were shown in Fig. 8. In consideration with transmission line theory the following relation was used to calculate Reflection loss^80^5
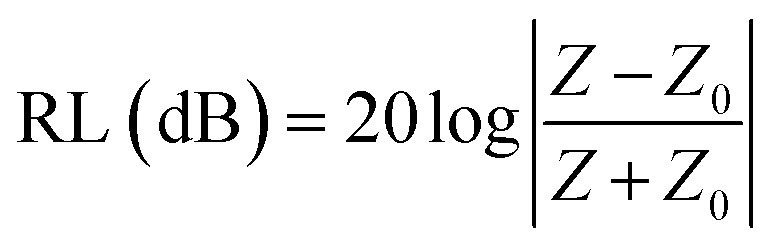
where *Z*_0_ is the impedance of free space and *Z* is the normalized input impedance of the absorber layer given by the relation6
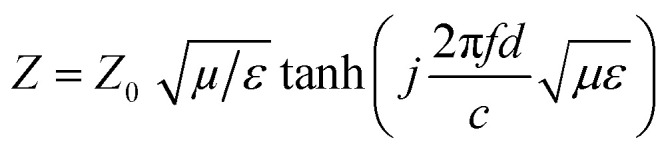
where, *ε*-complex permittivity, *µ*-complex permeability, *f*-frequency, *d*-absorber thickness, *c*-velocity of light.

The reflection loss (RL) spectra of SrDy_*x*_Ce_*x*_Fe_12−2*x*_O_19_ reveal a clear composition-dependent improvement in microwave absorption after Dy–Ce co-substitution. At the fixed absorber thickness of 3 mm, the undoped sample (*x* = 0.00) shows relatively weak attenuation in the X-band, with RL values remaining above the commonly accepted −10 dB threshold for efficient absorption. This indicates that pristine SrFe_12_O_19_, although magnetically active, does not possess a sufficiently balanced combination of dielectric loss, magnetic loss, and impedance matching to achieve strong microwave attenuation under the present measurement conditions.^81^

A marked enhancement in absorption performance is observed after rare-earth co-substitution. As the Dy–Ce concentration increases from *x* = 0.00 to *x* = 0.04, the RL minimum becomes progressively more negative, showing that moderate substitution improves the ability of the material to couple with and dissipate incident electromagnetic waves. In particular, compositions that cross the −10 dB level can be regarded as effective absorbers, since this criterion corresponds to more than 90% attenuation of incident microwave power.^82^ Among all investigated compositions, the sample with *x* = 0.04 exhibits the best absorption performance, reaching a minimum RL of −25.26 dB at approximately 9.6 GHz. This result identifies *x* = 0.04 as the optimum composition within the present series.

The increased RL response of the *x* = 0.04 sample is in agreement with the structural and magnetic modifications induced by the Dy–Ce substitution. The lattice distortion and defect sites are introduced by Dy–Ce substitution due to the difference of ionic radius and electronic configuration compared to Fe^3+^. These structural changes are in agreement with enhanced interfacial polarization and dielectric relaxation.^83–85^ At the same time, the change in magnetic anisotropy should influence the resonance-related processes of magnetic loss in the X-band frequency range.^86,87^ At *x* = 0.04, the effects are strong enough to increase the electromagnetic attenuation while keeping the phase purity, which provides a good balance between the wave entry and the energy dissipation and therefore the strongest reflection loss. In ferrite-based absorbers, strong attenuation generally requires incident microwaves to first enter the material efficiently and then undergo sufficient internal dissipation. The pronounced RL minimum observed at *x* = 0.04 therefore indicates that moderate Dy–Ce substitution most effectively tunes this balance.

At higher substitution levels, the absorption performance declines slightly. Although the compositions *x* = 0.08 and *x* = 0.10 still show improved RL compared with the undoped sample, their absorption intensity is weaker than that of *x* = 0.04. This behavior suggests that excessive substitution does not lead to a further improvement in microwave attenuation, but instead begins to disturb the optimal interplay between structural stability and electromagnetic loss processes. A similar optimum-at-intermediate-composition trend has also been reported in related rare-earth co-substituted hexaferrite systems.^64^

A particularly important result is observed for the sample *x* = 0.06, where the RL minimum shifts toward higher frequency and the absorption response becomes less favorable in the lower-frequency part of the X-band. This behavior is consistent with the XRD results, which indicate the presence of a secondary α-Fe_2_O_3_ phase at this composition. The appearance of an impurity phase can disrupt the electromagnetic homogeneity of the absorber, alter local dielectric and magnetic responses, and weaken the impedance balance required for effective microwave entry into the material. The *x* = 0.06 result therefore highlights the importance of phase purity in achieving strong and well-positioned microwave absorption. Comparison of the MW parameters is given in Table 4.

**Table 4:** Comparison of the reflection loss values of Dy–Ce substituted strontium hexaferrite with earlier research

Material	Method of preparation	Reflection loss (dB)	Ref.
Sr_1−*x*_Y_*x*_Fe_12_O_19_	Sol–gel method	−18.91	88
SrCoZnFe_16_O_27_	Ball milling	−19.5	89
SrFe_9_Mn_1·5_Ti_1·5_O_19_	Solid state method	−26	90
SrFe_(12−2*x*)_Co_*x*_Ti_*x*_O_19_	Ceramic method	−21.3	91
PPy/SrEr_0·3_Fe_11.7_O_19_	*In situ* polymerization	−24.01	92
Silver-doped strontium	Co-precipitation	−21.95	93
Zr–Zn–Co substituted strontium	Sol–gel method	−20	94
BaDy_*x*_Ce_*x*_Fe_12−2*x*_O_19_	Sol–gel method	−17.64	64
**SrDy**_**0.04**_ **Ce**_**0·04**_ **Fe**_**11.92**_ **O**_**19**_	**Sol–gel method**	**−25.26**	**This work**

**Fig. 9:**
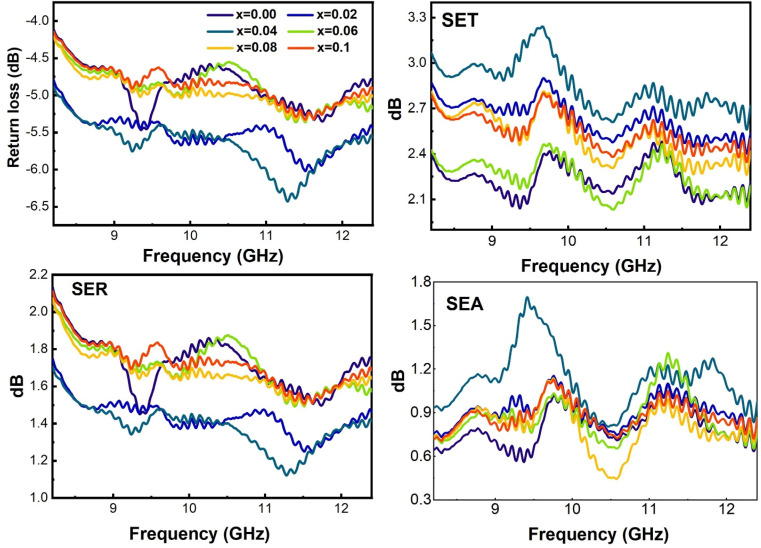
Variation of return loss, total shielding effectiveness (SET), shielding by reflection (SER) and shielding by absorption (SEA) of all samples of SrDy_*x*_Ce_*x*_Fe_12−2*x*_O_19_.

Electromagnetic shielding refers to the attenuation of incident electromagnetic radiation by a material through a combination of reflection, absorption, and, where relevant, multiple internal reflections.^95^ In the present work, the EMI shielding behavior of Dy–Ce co-substituted strontium hexaferrite was evaluated through the frequency-dependent variation of return loss, shielding effectiveness due to reflection (SER), shielding effectiveness due to absorption (SEA), and total shielding effectiveness (SET), as shown in Fig. 9.^96^ These parameters together provide a more complete picture of how the material interacts with incident microwaves than reflection loss alone, since they distinguish surface reflection from intrinsic dissipation within the absorber.

Across all compositions, the measured negative RL values in the X-band confirm that the incident electromagnetic waves interact effectively with the ferrite-based absorber. Among the investigated samples, the composition *x* = 0.04 exhibits the most favorable response, with the highest magnitude of return loss, approximately −6.5 dB near 11 GHz. This corresponds to attenuation of a substantial fraction of the incident microwave power and indicates that moderate Dy–Ce substitution improves coupling between the material and the incoming electromagnetic wave. Such behavior is generally associated with a more balanced interplay between dielectric and magnetic loss contributions, which enhances microwave entry into the material and promotes subsequent dissipation.^85^

A clearer understanding of the shielding mechanism is obtained from the SER and SEA components. The SER values remain in the range of approximately 1.2–2.1 dB for all compositions, showing that reflection contributes only moderately to the overall shielding response. The undoped SrFe_12_O_19_ sample exhibits comparatively higher SER, which suggests stronger impedance mismatch at the air-material interface and therefore greater reflection of incident microwaves from the surface. After Dy–Ce substitution, SER decreases, indicating that the substituted compositions possess improved impedance compatibility with free space. In particular, the lowest SER observed for *x* = 0.04 implies that this composition allows more efficient entry of electromagnetic waves into the material rather than rejecting them at the surface.

In contrast, the shielding effectiveness due to absorption, SEA, is the dominant contribution throughout the composition series. The increase in SEA after substitution demonstrates that Dy–Ce incorporation enhances the intrinsic dissipation of electromagnetic energy within the ferrite matrix. The highest SEA, approximately 1.7 dB around 9.4 GHz, is again observed for the *x* = 0.04 composition, confirming that this sample offers the most favorable balance between wave penetration and internal attenuation. This absorption-dominant behavior is particularly desirable from a materials-design perspective because it reduces secondary reflection and indicates that attenuation occurs primarily inside the material rather than only at its surface.

The total shielding effectiveness, SET, obtained as the combined contribution of reflection and absorption, lies in the range of about 2.0–3.3 dB for the investigated compositions, with the maximum value of approximately 3.2 dB also observed for *x* = 0.04. Although these SET values are modest in absolute terms, the composition-dependent trend is still meaningful: Dy–Ce co-substitution clearly improves the shielding response relative to pristine SrFe_12_O_19_, and the improvement arises predominantly from enhanced absorption rather than increased reflection. This result is consistent with the reflection loss analysis and with the broader electromagnetic characterization presented in subsequent sections, which together indicate that the optimized composition achieves a more favorable dielectric–magnetic balance.

It is worth noting that even though Dy–Ce co-substitution results in better shielding effectiveness than pristine SrFe_12_O_19_, the SET values are still relatively low. Such values are associated with partial attenuation of incident electromagnetic radiation and are lower than those generally needed for high-performance EMI shielding applications.^97,98^ The present materials should not be regarded as standalone shielding materials for demanding electromagnetic protection environments. Nonetheless, the observed composition-dependent enhancement confirms the fact that the substitution of rare-earths can be an effective way to improve the electromagnetic response of SrFe_12_O_19_ and provides useful insight into the mechanisms of microwave attenuation. The shielding effectiveness can be further enhanced by microstructural optimization, varying the thickness of the absorber, making composites with conductive phases or hybridizing the absorber with dielectric materials that can provide attenuation and impedance matching.^99,100^

#### 3.5.2 Attenuation constant and eddy current losses

**Fig. 10:**
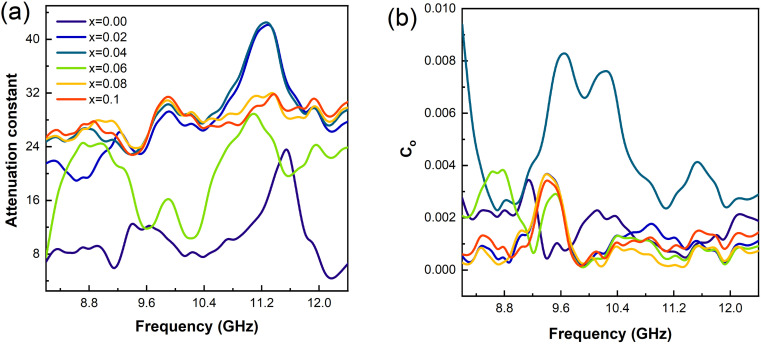
Variation of (a) attenuation constant and (b) eddy current losses with frequency for all samples of Dy–Ce substituted SrFe_12_O_19_ nanoparticles.

Fig. 10a and b present the frequency-dependent variation of the attenuation constant and eddy current coefficient for Dy–Ce co-substituted SrFe_12_O_19_ in the X-band region. The attenuation constant is an important parameter for evaluating microwave absorbers because it reflects the ability of a material to dissipate incident electromagnetic energy once the wave enters the absorber. In ferrite-based systems, this attenuation behavior is governed by the combined contribution of dielectric and magnetic responses, and therefore depends strongly on the frequency-dependent complex permittivity and permeability of the material.^101^

Among all compositions, the undoped sample (*x* = 0.00) exhibits the lowest attenuation constant throughout the measured frequency range, indicating limited intrinsic capacity for microwave dissipation. After Dy–Ce co-substitution, the attenuation constant increases noticeably, demonstrating that rare-earth incorporation improves the electromagnetic energy-loss capability of SrFe_12_O_19_. The composition *x* = 0.04 shows the highest attenuation constant, with a pronounced enhancement in the range of approximately 10.5–11.5 GHz. This result is consistent with the reflection loss and shielding analyses discussed earlier, and further confirms that moderate Dy–Ce substitution creates the most favorable conditions for microwave attenuation in the present system. The better attenuation behavior of *x* = 0.04 is consistent with its better impedance matching, better dielectric loss and better magnetic loss properties. These factors are consistent with increased dissipation of electromagnetic energy in the absorber and decreased reflection of incident microwaves.^102,103^

To further analyze the magnetic dissipation behavior, the eddy current coefficient *C*_0_ was examined as a function of frequency. In principle, if magnetic loss arises predominantly from eddy current effects, *C*_0_ should remain nearly constant over the frequency range under consideration. By contrast, frequency-dependent variation in *C*_0_ generally indicates that other magnetic loss processes, such as natural ferromagnetic resonance or exchange resonance, also contribute to the overall attenuation.^104^ In the present study, the *C*_0_ curves are not strictly frequency independent, and instead show noticeable deviations and fluctuations across the X-band. This behavior indicates that the magnetic loss mechanism in Dy–Ce co-substituted strontium hexaferrite cannot be attributed solely to eddy current dissipation.

Rather, the observed response suggests that microwave attenuation in these samples originates from a combination of magnetic loss processes. The coexistence of eddy current loss with resonance-related mechanisms is reasonable for substituted hexaferrites, where cation substitution, lattice distortion, grain growth, and magnetic anisotropy can collectively influence the resonance condition and the dissipation pathway of the incident microwave field. The more pronounced *C*_0_ response for the substituted samples, particularly for *x* = 0.04, therefore indicates enhanced magnetic-loss activity overall, but not exclusive dominance of the eddy current mechanism.

This interpretation is important because it aligns more closely with the broader electromagnetic data presented in the manuscript. The optimized composition *x* = 0.04 not only exhibits the highest attenuation constant, but also shows improved reflection loss, greater absorption contribution, and more favorable magnetic and dielectric loss characteristics. The attenuation behavior observed in Fig. 10 can therefore be understood as part of a cooperative electromagnetic response, in which Dy–Ce co-substitution enhances the internal dissipation of microwave energy through multiple interacting mechanisms. In contrast, the lower attenuation constant of the undoped sample confirms that pristine SrFe_12_O_19_ lacks the same level of loss optimization and is therefore less effective as an X-band microwave attenuator under the present conditions.

#### 3.5.3 Complex permittivity and permeability

**Fig. 11:**
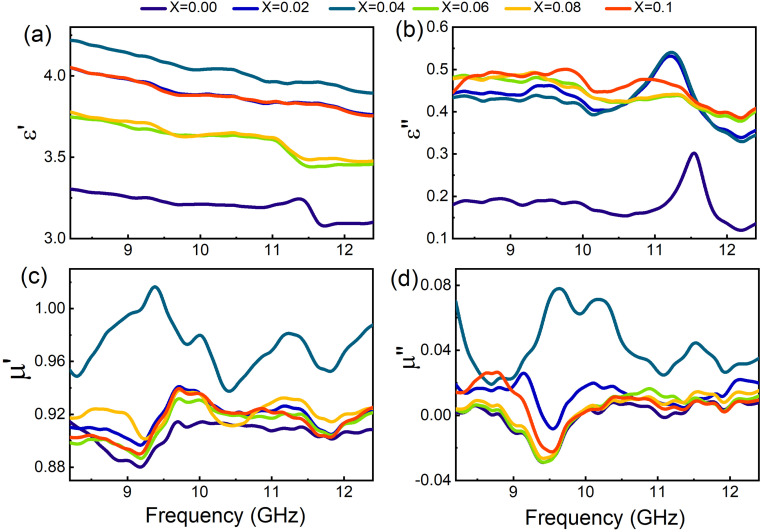
Variation of electromagnetic characteristics real and imaginary permittivity (a & b) and permeability (c & d) with frequency.

The frequency-dependent electromagnetic parameters of SrDy_*x*_Ce_*x*_Fe_12−2*x*_O_19_, namely the real and imaginary parts of complex permittivity (*ε*′, *ε*″) and complex permeability (*µ*′, *µ*″), are presented in Fig. 11 for all compositions *x* = 0.0–0.1. These parameters are of central importance in microwave absorption analysis because *ε*′ and *µ*′ describe the capacity of the material to store electric and magnetic energy, whereas *ε*″ and *µ*″ represent the corresponding dissipation channels responsible for dielectric and magnetic loss.

For all compositions, the real part of permittivity, *ε*′, gradually decreases with increasing frequency. This dispersion behavior is typical of ferrite-based systems and is generally associated with the progressive inability of space-charge carriers and interfacial dipoles to follow the rapidly alternating electromagnetic field at higher frequencies.^105,106^ At lower frequencies, these polarization mechanisms can respond more effectively, leading to higher values of *ε*′. As the frequency increases into the X-band, the delayed response of these polarization entities results in a reduction of the real permittivity. The observed trend therefore reflects the expected dielectric relaxation behavior of substituted hexaferrites rather than any structural instability.

The imaginary part of permittivity, *ε*″, which represents dielectric energy dissipation, exhibits composition-dependent enhancement and shows noticeable features in the 10–11 GHz region. Such behavior indicates the presence of frequency-dependent relaxation processes and suggests increased dielectric loss in the substituted samples. In ferrite materials, this type of dielectric response is often associated with interfacial polarization and localized charge hopping, including electron exchange between mixed-valence iron ions such as Fe^2+^/Fe^3+^.^107^ The increased values of *ε*″ after Dy–Ce substitution are in agreement with increased interfacial polarization, defect induced dipoles and localized charge hopping between iron ions.^108^ Also, local charge imbalance and defect states in the hexaferrite lattice can arise due to Dy–Ce substitution, primarily due to the mixed valence states of cerium.^11^ The structural and electronic heterogeneities offer additional polarization centers and increase the distribution of relaxation times, which promote dielectric relaxation and energy dissipation under alternating electromagnetic fields.^109^ The observed dielectric response can be understood by an increase of the number of polarization centers and a broader distribution of relaxation times upon Dy–Ce substitution leading to enhanced dielectric energy dissipation. From the microwave absorption standpoint, this is an important result because dielectric loss contributes directly to attenuation once the wave enters the absorber.

The magnetic response also shows systematic variation with substitution. The real part of permeability, *µ*′, remains close to unity with only modest frequency dependence across the X-band. This behavior is characteristic of ferrite absorbers operating in the microwave region, where domain-wall motion is largely suppressed and magnetic response arises primarily from spin-related processes rather than low-frequency wall displacement.^10^ The relatively stable *µ*′ values indicate that the samples maintain a measurable magnetic energy-storage capability within the investigated frequency range, while avoiding the strong low-frequency magnetic dispersion typical of softer magnetic systems.

More importantly, the imaginary part of permeability, *µ*″, shows distinct peaks and enhanced magnitude for the Dy–Ce-substituted compositions, indicating improved magnetic loss.^110^ In hexaferrite-based absorbers, *µ*″ is commonly associated with resonance-related magnetic dissipation, including spin relaxation and natural ferromagnetic resonance. The increase in *µ*″ after Dy–Ce substitution is a signature of more magnetic energy dissipation because of the change in magnetic anisotropy and spin dynamics of the hexaferrite lattice.^111^ Rare-earth ions can modify the local magnetic interactions and the resonance conditions, thus enhancing the magnetic loss processes related to the resonance in the X-band region.^112^ This is consistent with the earlier attenuation constant analysis, which showed that the substituted samples, especially *x* = 0.04, possess a stronger capacity to dissipate incident microwave energy. The enhancement in *µ*″ is thus not an isolated observation, but part of the broader trend showing that moderate rare-earth substitution improves the microwave-loss characteristics of SrFe_12_O_19_.

A key outcome of Fig. 11 is that the substituted samples show simultaneous improvement in both dielectric loss (*ε*″) and magnetic loss (*µ*″). This balanced dielectric–magnetic dissipation is highly desirable for microwave absorption because efficient absorbers generally require not only strong internal loss mechanisms, but also a suitable balance between electric and magnetic responses to support impedance matching.^113^ In the present system, Dy–Ce substitution appears to tune both contributions in a cooperative manner, thereby improving the overall attenuation behavior. This interpretation is fully consistent with the reflection loss, shielding, and attenuation constant results, all of which identify the moderately substituted compositions, particularly *x* = 0.04, as the most effective microwave attenuators in the series.

#### 3.5.4 Tangent losses

**Fig. 12:**
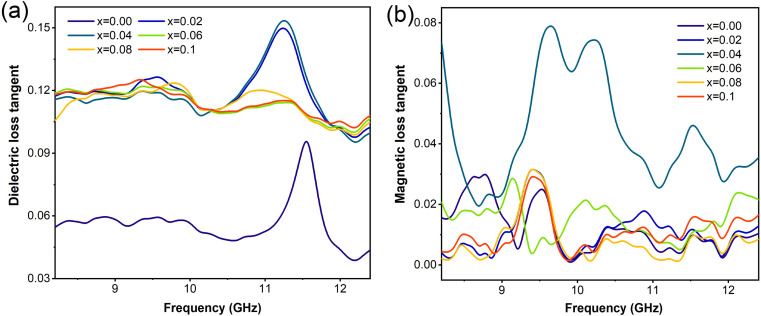
Variation of (a) tan *δ*_*ε*_ and (b) tan *δ*_*µ*_ with frequency for all samples of Dy–Ce substituted SrFe_12_O_19_ nanoparticles.

The frequency-dependent dielectric loss tangent (tan *δ*_*ε*_) and magnetic loss tangent (tan *δ*_*µ*_) of Dy–Ce co-substituted Sr-hexaferrite are shown in Fig. 12a and b, respectively. These parameters are useful for evaluating microwave absorption because they directly reflect the relative ability of the material to dissipate electric and magnetic energy under an alternating electromagnetic field. In general, an increase in either loss tangent indicates enhanced energy-conversion capability within the absorber matrix.

The undoped sample (*x* = 0.00) exhibits comparatively low dielectric loss tangent over the entire X-band, indicating limited dielectric dissipation. After Dy–Ce co-substitution, tan *δ*_*ε*_ becomes more pronounced, while still showing only moderate variation with frequency. A noticeable enhancement is observed near 11.5 GHz, which can be attributed to frequency-dependent dielectric relaxation associated with dipolar polarization and space-charge effects.^114^ This behavior implies that the substitution of the rare-earth ions introduces additional polarization centers that are attributed to lattice distortion and defect formation, and that are responsible for the enhancement of dielectric relaxation processes and increased dielectric energy dissipation.

A similar composition-dependent improvement is observed in the magnetic loss tangent. The undoped SrFe_12_O_19_ sample shows relatively weak tan *δ*_*µ*_, indicating limited magnetic dissipation under the present measurement conditions. In contrast, the substituted samples display higher magnetic loss tangent values, with the most pronounced response observed for the composition *x* = 0.04, where tan *δ*_*µ*_ reaches approximately 0.07–0.08. These microstructural changes are in good agreement with the observed composition-dependent variations in magnetic and microwave attenuation behavior discussed in the following sections. The simultaneous enhancement of tan *δ*_*ε*_ and tan *δ*_*µ*_ is particularly important for understanding the optimized microwave absorption behavior of the substituted samples. Efficient absorbers generally require not only strong dielectric or magnetic loss individually, but also a suitable balance between the two so that incident microwaves can enter the material and then be effectively dissipated. In the present system, the composition *x* = 0.04 exhibits the most favorable combination of enhanced dielectric loss, appreciable magnetic loss, and frequency-dependent loss behavior, which is fully consistent with its superior reflection loss and attenuation characteristics discussed in the previous sections. Therefore, the tangent-loss analysis further supports the conclusion that moderate Dy–Ce co-substitution improves the dielectric–magnetic loss balance of SrFe_12_O_19_ and thereby enhances its X-band microwave attenuation performance.

#### 3.5.5 Cole–Cole analysis

**Fig. 13:**
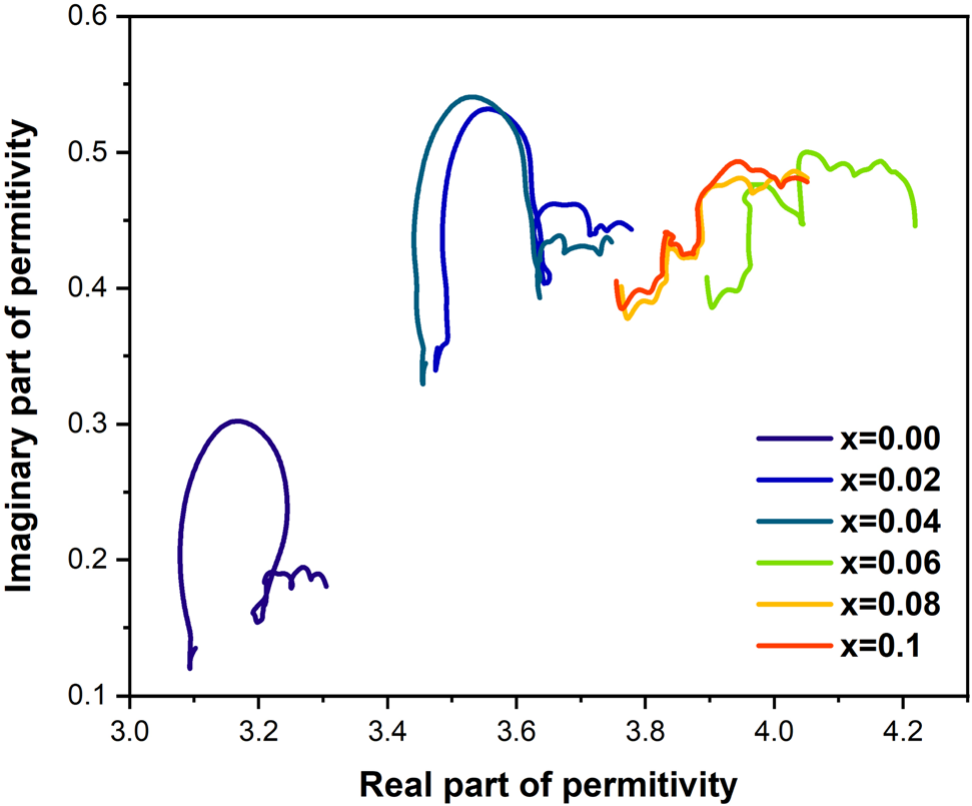
Cole–Cole plots of SrDy_*x*_Ce_*x*_Fe_12−2*x*_O_19_ (*x* = 0.00, 0.02, 0.04, 0.06, 0.08, 0.1) in X band.

The dielectric relaxation behavior of Dy–Ce co-substituted SrFe_12_O_19_ was further examined using Cole–Cole plots, constructed from the relationship between the real (*ε*′) and imaginary (*ε*″) parts of complex permittivity, as shown in Fig. 13.^115^ Cole–Cole analysis is widely used to assess polarization behavior in microwave-absorbing materials because it provides insight into whether dielectric relaxation follows an ideal single-relaxation Debye process or a more distributed non-ideal response.

For the undoped sample (*x* = 0.0), the Cole–Cole plot exhibits only a small and somewhat distorted semicircular feature, indicating relatively weak dielectric relaxation. In contrast, the Dy–Ce-substituted samples show larger depressed semicircular arcs together with distorted loop-like characteristics. According to Debye theory, an ideal semicircle corresponds to a single relaxation process governed by one characteristic relaxation time.^116^ The deviation from a perfect semicircle observed here therefore suggests that the substituted samples do not follow ideal Debye behavior, but instead exhibit distributed relaxation associated with multiple polarization processes operating simultaneously.^117^

Such a non-Debye relaxation implies that the Dy–Ce substitution induces a broad distribution of polarization environments as a result of the lattice distortion, defect formation and interfacial heterogeneity. These features provide multiple relaxation pathways leading to dielectric loss over a wider frequency range. In the present case, Dy–Ce incorporation appears to increase the complexity of the dielectric response by creating a broader distribution of relaxation times within the material. This interpretation is consistent with the earlier permittivity analysis, where enhanced *ε*″ values and frequency-dependent dielectric loss were observed for the substituted compositions. Thus, the Cole–Cole plots provide additional evidence that Dy–Ce substitution strengthens dielectric attenuation through more complex polarization and relaxation processes.

The larger and more prominent depressed arcs observed for the intermediate compositions suggest that these samples possess a more favorable balance between polarization strength and relaxation dynamics. In particular, the compositions that exhibit more prominent Cole–Cole features are indicative of a higher contribution of dielectric relaxation processes, which is consistent with improved dielectric energy dissipation in the measured frequency range. Rather than interpreting non-Debye relaxation as an isolated result, it is more appropriate to view it as part of the broader electromagnetic response of the material, where distributed dielectric relaxation works together with magnetic loss and impedance matching to improve microwave attenuation.

#### 3.5.6 Impedance matching

**Fig. 14:**
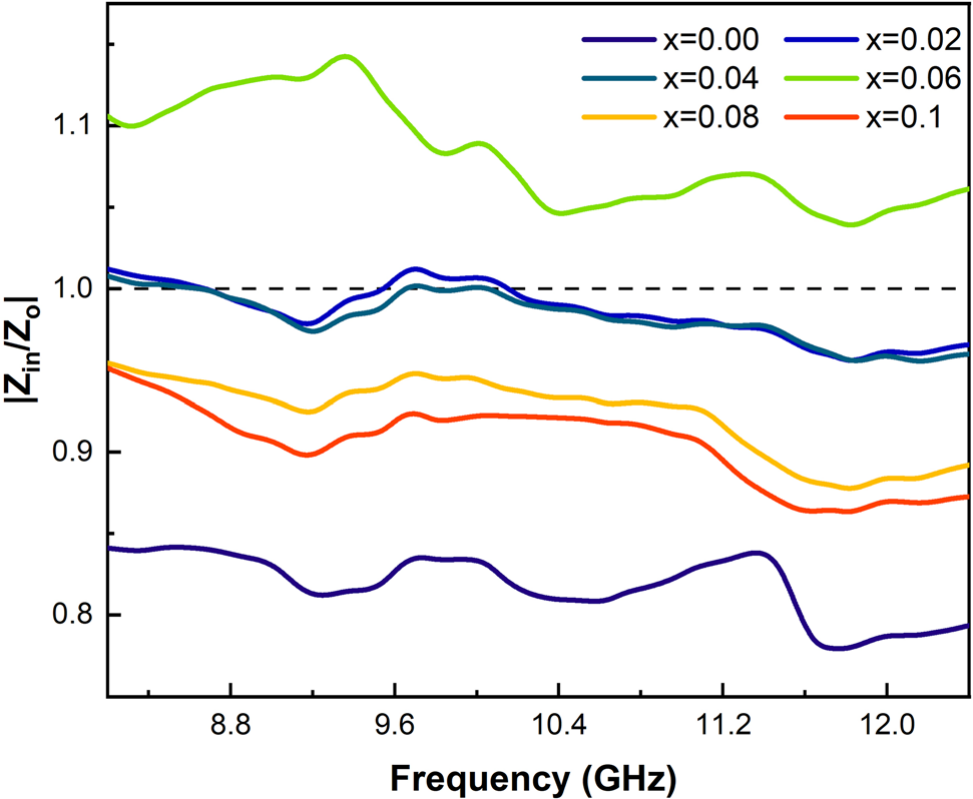
Impedance matching of SrDy_*x*_Ce_*x*_Fe_12−2*x*_O_19_ (*x* = 0.00, 0.02, 0.04, 0.06, 0.08, 0.1) in X band.

Fig. 14 shows the frequency-dependent normalized input impedance, |*Z*_in_/*Z*_0_|, of SrDy_*x*_Ce_*x*_Fe_12−2*x*_O_19_ in the X-band for different substitution levels (*x* = 0.00–0.10). Impedance matching is a critical parameter in microwave absorption because efficient attenuation requires incident electromagnetic waves to first enter the absorber with minimal reflection at the air–material interface.^118^ In an ideal case, |*Z*_in_/*Z*_0_| approaches unity, indicating that the intrinsic electromagnetic parameters of the absorber are well matched to free space and that more of the incident wave can propagate into the material for subsequent dissipation.^87–93^

The undoped sample (*x* = 0.00) exhibits |*Z*_in_/*Z*_0_| values significantly lower than unity throughout the measured frequency range, indicating pronounced impedance mismatch. This result is consistent with its weak reflection-loss performance and confirms that pristine SrFe_12_O_19_ does not provide a sufficiently balanced dielectric–magnetic response for efficient microwave entry under the present conditions. In such a case, a considerable portion of the incident electromagnetic wave is reflected at the material surface before effective internal attenuation can occur.

After Dy–Ce co-substitution, the impedance spectra move progressively closer to unity, indicating a systematic improvement in impedance matching. This trend shows that the dielectric and magnetic responses of the material are simultaneously tuned by Dy–Ce substitution, which reduces the mismatch of electromagnetic parameters between the absorber and free space. This provides a better impedance match, so that a larger fraction of incident microwaves can enter the absorber and interact with the internal loss mechanisms. Among all compositions, the sample with *x* = 0.04 exhibits |*Z*_in_/*Z*_0_| values closest to unity over a relatively broad frequency range of approximately 8.8–10.0 GHz. This behavior indicates that the electromagnetic parameters of this composition are most favorably balanced for microwave absorption, allowing incident waves to enter the material more efficiently and undergo stronger internal attenuation.

The impedance-matching result for *x* = 0.04 is fully consistent with the other electromagnetic findings reported in this work. The same composition also exhibits the strongest reflection loss, the highest attenuation constant, enhanced dielectric and magnetic loss components, and the most favorable absorption-dominant shielding response. This consistency is important because strong microwave absorption cannot be attributed to internal loss processes alone; it requires simultaneous optimization of wave entry and wave dissipation. The near unity |*Z*_in_/*Z*_0_| behavior of the *x* = 0.04 sample therefore provides a key explanation for why this composition shows the best overall microwave attenuation performance in the present series.

At higher substitution levels, the impedance response remains improved relative to the undoped sample but becomes less optimal than that of *x* = 0.04. This suggests that excessive substitution begins to disturb the dielectric–magnetic balance required for ideal matching, even if some enhancement in loss mechanisms is still retained. In other words, Dy–Ce co-substitution is beneficial only within an appropriate compositional window, beyond which the gain in electromagnetic loss is offset by deterioration in structural or interfacial conditions that influence matching.

The enhanced microwave absorption performance of the *x* = 0.04 composition corresponds to the simultaneous optimization of impedance matching, dielectric loss, magnetic loss, and attenuation capability. From a microscopic point of view, the Dy–Ce substitution introduces local structural heterogeneity due to lattice distortion and defect formation, and also modifies magnetic interactions within the hexaferrite lattice. These effects lead to dielectric dissipation associated with polarization and magnetic loss associated with resonance, and result in more effective attenuation of electromagnetic energy. These microscopic contributions, along with the observed favorable impedance matching of this composition, are responsible for its excellent microwave absorption performance.

## 4. Conclusions

Dy–Ce co-substituted strontium hexaferrite, SrDy_*x*_Ce_*x*_Fe_12−2*x*_O_19_, was successfully synthesized by the sol–gel auto-combustion route and systematically investigated to establish the relationship between composition, structure, magnetic behavior, and microwave attenuation performance. Rietveld analysis showed that the M-type hexaferrite structure was retained over most of the composition range, with only limited lattice distortion after substitution, although the appearance of α-Fe_2_O_3_ at *x* = 0.06 indicated partial phase instability at this composition. FT-IR and electron microscopy further supported the preservation of the hexaferrite framework and revealed substitution-driven microstructural evolution toward larger platelet-like grains. Magnetic measurements showed that moderate Dy–Ce substitution improved the ferrimagnetic response, with *x* = 0.04 exhibiting the most favorable combination of saturation magnetization and magnetic-loss-related behavior, whereas higher substitution levels reduced magnetic performance due to disorder and phase-related effects. Electromagnetic characterization confirmed that Dy–Ce co-substitution enhances the microwave attenuation capability of SrFe_12_O_19_ by improving dielectric loss, magnetic loss, attenuation constant, and impedance matching. Among all compositions, *x* = 0.04 delivered the best microwave absorption performance, with a minimum reflection loss of −25.26 dB at 9.6 GHz for a 3 mm absorber thickness. The shielding response was found to be absorption dominant, although the absolute shielding effectiveness remained limited, indicating that the developed material is better described as an absorption-oriented microwave attenuator than as a high-performance bulk EMI shield. Overall, the results show that controlled Dy–Ce co-substitution provides an effective route for tuning the dielectric–magnetic balance of SrFe_12_O_19_ and for optimizing its X-band microwave absorption behavior.

## Conflicts of interest

There are no conflicts to declare.

## Supplementary Material

RA-OLF-D6RA02983C-s001

## Data Availability

Data will be made available upon request. Supplementary information (SI) is available. See DOI: https://doi.org/10.1039/d6ra02983c.
