# Supplementary material for: Tuning the electromagnetic and magnetic response of SrFe12O19 by Dy–Ce co-substitution for X-band microwave absorption
Source: RSC Adv. 2026 Jul 3;16(35):35400–17. doi: 10.1039/d6ra02983c (PMC13329756; doi:10.1039/d6ra02983c)
Supplement: RA-016-D6RA02983C-s001 [file RA-016-D6RA02983C-s001.pdf]

Supplementary Information (SI) for RSC Advances.
This journal is © The Royal Society of Chemistry 2026

Supporting Information

Tuning the Electromagnetic and Magnetic Response of $SrFe_{12}O_{19}$ by Dy-Ce Co-Substitution for X-Band Microwave Absorption

Shreepad S. Atkare[1], S. B. Deshmukh [2], Vinod N. Dhage [3,] Akash V. Fulari[4], Heba Taha Mohmmed Abdelghani,[5,]* Sagar E. Shirsath [6,]*, Maheshkumar L. Mane[1,]*

[1]Shikshan *Maharshi Guruvarya R. G. Shinde Mahavidyalaya, Paranda, Osmanabad, MS, India*
[2] *Department of Physics, Ramkrishna Paramhans College, Dharashiv, MS, India*
[3]*Advanced Materials and Nanotechnology Research Laboratory, Department of Physics, MES Abasaheb Garware College, Pune 411004, MS, India*
[4] *Symbiosis Centre for Research and Innovation, Symbiosis International (Deemed University), Pune, MS, India*
[5] *Department of Exercise Physiology, College of Sport Sciences and Physical Activity, King Saud University, P.O. Box 2454, Riyadh 11451, Saudi Arabia*
[6] *School of Materials Science and Engineering, University of New South Wales, Sydney, NSW 2052, Australia*

Table 1: Quantity of precursors used for the preparation of 10gm of materials of $SrDy_xCe_xFe_{12-2x}O_{19}$ (x=0.00, 0.02, 0.04, 0.06, 0.08, 0.1) using sol gel method

**Quantity of precursors used (gm)**					
**Comp. (x)**	**SrN_2O_6**	**$Dy(NO_3)_3$ $XH_2O)$**	**$(Ce(NO_3)_3$ $6H_2O)$**	**$(FeN_3O_9.$ $9H_2O)$**	**$(C_6H_8O_7.$ $H_2O)$**
0.00	1.993	0.000	0.000	45.660	5.437
0.02	1.986	0.065	0.082	45.346	5.418
0.04	1.979	0.130	0.162	45.033	5.398
0.06	1.972	0.195	0.243	44.722	5.379
0.08	1.965	0.259	0.323	44.413	5.360
0.1	1.958	0.322	0.402	44.107	5.341
